# Three cases of discontinuous refractive index in metamaterial study

**DOI:** 10.1038/s41598-022-07537-1

**Published:** 2022-03-03

**Authors:** Antoine Wegrowski, Wei-Chih Wang, Chileung Tsui

**Affiliations:** 1 Department of Mechanical Engineering, University of Washington, 165 Stevens Way, Box 352600, Seattle, WA 98195 USA; 2 Department of Electrical Engineering, University of Washington, 185 Stevens Way, 352500, Seattle, WA 98195 USA; 3 Department of Power Mechanical Engineering, National Tsing Hua University, 101, Section 2, Kuang-Fu Road, Hsinchu, 30013 Taiwan, ROC; 4 Institute of Nano Engineering and Microsystems, National Tsing Hua University, 101, Section 2, Kuang-Fu Road, Hsinchu, 30013 Taiwan, ROC

**Keywords:** Metamaterials, Electrical and electronic engineering, Terahertz optics

## Abstract

We investigate three cases of metamaterials presented in the literature displaying refractive index with one or more discontinuities along the frequency spectrum. We reproduce the numerical simulations of these metamaterials and compare our simulations to each reported case. For each case, we perform a geometrical investigation of each metamaterial’s refractive index by mean of a numerical simulation of a prism made of the reported metamaterials upon which is incident a plane electromagnetic wave. Such investigation allows us to infirm or confirm negative refraction at resonance frequency. Finally, we carry a numerical and theoretical investigation of this discontinuity and show that, as the refractive index crosses a discontinuity, while the topology of the effective wave has changed within the metamaterial, the dynamics of the phases remain unchanged at any time at the metamaterial's boundaries.

## Introduction

The propagation of electromagnetic waves in a given material are governed by two complex quantities characteristic of that material: the permittivity ε, and the permeability μ. These two quantities are used to define a third one, the refractive index n, given by the Maxwell relation:

1 $$ n = \sqrt {\varepsilon \mu } $$

This refractive index has been believed to be always positive, until 1968, when Veselago^1^ introduced the idea of a substance that can have a negative refractive index. He hypothesized a material whose permittivity ε and permeability μ are negative and demonstrated that, in such a case, n has its real part taking a negative value out of the two possible, opposed in sign, solutions of Eq. (1). Veselago showed that this result does not violate any law of physics, and described how familiar effects, such as the Doppler effect or the Vavilov–Cherenkov radiation, would be counterintuitively modified when taking place within negative refractive index materials (henceforth NIM). While Veselago suggested a way to produce such NIMs, his results received at the time very little attention, dismissed as theoretical fantasy, as no naturally occurring material presented this feature.

### Metamaterials

**Figure 1:**
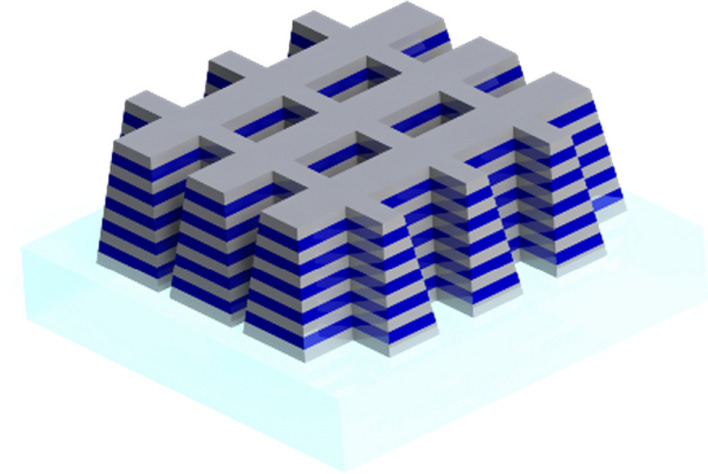
Fishnet metamaterial made of superposed layers of Au/Al2O3/Au.

It was not until 2000 that Smith et al.^2^ produced a composite medium displaying the double negative permittivity and permeability Veselago predicted. This material, by the fact that it was composite and displayed exotic characteristics, i.e. characteristics not found in nature, fitted the definition of metamaterials. This experimental realization caused an explosion of interest toward NIMs. A major figure in this initial impetus was John Pendry, who, a few months after Smith et al., suggested that a lens made of NIMs would beat the “natural" lenses resolution (i.e. no smaller than a wavelength)^3^, and in 2006 introduced, at the same time as, yet independently from, Ulf Leonhardt, the idea of a metamaterial-based invisibility cloak^4,5^. Such theoretical promises, quickly followed by experimental realizations^6–8^, reinforced the academic interest surrounding negative refraction. While the original design by Smith et al. was based on the so-called split ring resonator (SRR), a variety of designs appeared throughout the following years^9–17^. Most of the proposed NIMs, Zhang et al.'s fishnet included, are termed metasurfaces, presenting as films that can be laid on top of one another in order to create a layer of metamaterial of desired thickness^18^ (see Fig. 1). Another often recurring specificity of NIMs is their anisotropy: most NIMs only display negative refraction when the plane wave incident upon them propagates normally to the surface and is polarized along one specific axis. These constrain the shapes and uses for which metamaterials can be used. 3D metamaterials do exist^19–21^, but have been much more challenging to develop.

Another challenge is the limitations in transmission and frequency of operation of metamaterials. Most metamaterials developed so far are operable only in the infrared to microwave frequency range, as the development of negative refraction at optical frequencies meets serious technical challenges due to the dimensions constraints of the metamaterial's structure^22^. Metamaterials also display significant absorption around magnetic resonance; such absorption is often quantified using the Figure of Merit (FOM), calculated as the ratio of the real to imaginary part of the refractive index. Absorption is evidently an undesirable effect to any application of metamaterials: a metamaterial-based imagery device would reduce the quality of the resolved image, while an invisibility cloaking system, typically designed for stealth, could be detected by noticing the unusual absorption of the system's location.

### Effective electromagnetic parameters retrieval

One way to retrieve the NIM's effective electromagnetic parameters, and in particular the refractive index, requires a knowledge of the complex transmission and reflection coefficients of the wave incident to the material. Regardless of whether transmission and reflection obtained by experiment or numerical simulation, one needs a way to convert these to the optical parameters. Such an algorithm has been developed. In 1970, Nicolson et al.^23^ are the first to derive the set of equations relating transmission and reflection to refractive index and impedance, developing the well-known Nicolson–Ross–Weir (NRW) method. Chen et al.^24^ in turn improved this algorithm, allowing the first boundary and thickness of the effective metamaterial (those being different from the physical boundaries and thickness of the metamaterial, as the electromagnetic wave is usually affected ahead and after the physical boundaries by the metamaterial) as well as the sign of the effective impedance and the branch of the real part of the refractive index to be determined. More recently, Hsieh and Wang^25^ suggested another method applied to bianisotropic materials and based on material dispersion models. The most important assumption that the above algorithms rest upon is that of the material's homogeneity: the inhomogeneity within the material, mainly, its stratified composition and planar pattern, have to be designed within length scales that are very small compared to the effective wavelength of the wave propagating within the metamaterial (as an example, Ding et al.'s fishnet^14^ is constituted of 30 μm-thick unit cells, displaying negative refractive index for electromagnetic waves of wavelength 0.1 mm, three times larger).

In their article Chen et al. point out how, around resonance frequency, the wavelength within the material approaches the material's length scale, thereby excluding the possibility of a homogeneity assumption and making the situation one that is outside of the range of application of their algorithm. This is true inasmuch as the wavelength within a material equals the product of the wavelength in free space and the refractive index of the material: the larger the refractive index, the smaller the wavelength within the material. Regardless, this homogeneity assumption has often been disregarded in other papers^11,13–17^, who make the choice of presenting the results of the retrieval of effective electromagnetic parameters as accurate, even in the region of magnetic resonance, precisely where the wavelength of the radiation within the medium reduces to the same order of magnitude as the thickness of the material. The fact that the algorithm's hypothesis is not verified in the window of frequency where magnetic resonance occurs is tantalizing since this is precisely where the refractive index turns negative!

Smith et al.^26^ solved this problem by suggesting an algorithm to retrieve effective electromagnetic parameters in an inhomogeneous material. The^11,13–17^ papers referenced above, however, do not refer to this method and insist on wrongly using Chen et al.'s algorithm. One more algorithmic challenge, to which Smith et al. has not brought an answer, is that of what we will hence-forth refer to as the m-branch index jump. In the above algorithms, the expression of the refractive index contains an inverse cosine, and is therefore dependent on an associated m-branch, as can be seen in Eq. (2):

2 $$ n = \frac{1}{kd}cos^{ - 1} \left[ {\frac{1}{{2S_{21} }}\left( {1 - S_{11}^{2} + S_{21}^{2} } \right)} \right] + \frac{2\pi m}{{kd}} $$

The determination of such m-branch index is usually done using physical requirements on the system or by a requirement that the real part of the refractive index be continuous. Some metamaterials^16,17,27^, however, have been reported to display a discontinuous refractive index, jumping from positive to negative, i.e. with the m-branch index jumping from one value to another. No consideration of this case seems to have been done when developing the above algorithms, leaving the decision of the frequency at which the m-branch index jump occur up to the arbiter of the researchers, such as in^16^. Some papers^27^ do acknowledge the limits of the algorithm and the impossibility to decide where the jump occurs.

### Proposed solution

In this article, we present three cases of metamaterials presented in the literature displaying refractive index with one or more discontinuities along the frequency spectrum. We reproduce the numerical simulations of these metamaterials and compare our simulations to each reported case. For each case, we perform a geometrical investigation of each metamaterial's refractive index by mean of a numerical simulation of a prism made of the reported metamaterials upon which is incident a plane electromagnetic wave. Such investigation allows us to infirm or confirm negative refraction at resonance frequency. Finally, we carry a numerical and theoretical investigation of this discontinuity and show that, as the refractive index crosses a discontinuity, while the topology of the effective wave has changed within the metamaterial, the dynamics of the phases remain unchanged at any time at the metamaterial's boundaries.

## Three cases of discontinuous refractive index

In this section, we investigate what appears to be an anomaly of the NRW method: the discontinuity of the refractive index in some materials. We will first consider three designs presented in the literature, presenting their characteristic in details. We will then offer an alternative way to measure the refractive index of metamaterials, and suggest a possible explanation for the emergence of the discontinuity. We have identified three designs reported in the literature, which present discontinuous refractive index. Numerical simulation of these metamaterials is performed using the commercial software CST Microwave Studio^30^ and results are compared to those of the respective reports, in order to confirm the faithfulness of our numerical reproduction of those metamaterials. In each case, a unit cell of metamaterial is built on the software, lying in the XY plane. Boundaries are set as magnetic (H_t_ = 0) along the X-axis and electric (*E*_*t*_ = 0) along the Y axis, with open boundaries along the Z axis, axis of propagation of the plane wave. A plane wave polarized along the Y axis is sent for normal incidence upon the unit cell, and the S-parameters are retrieved for analysis using the NRW method. Meshing is carefully monitored to always exceed 50,000 tetrahedrons.

### Chan et al.'s arrays of upright split-ring pairs

Chan et al.^17^ proposed a metamaterial operating in near-infrared frequency, consisting in split-ring pairs arranged back to back. The split-ring pair is made of gold (Drude permittivity of $\varepsilon = 9 - \frac{{\omega_{p}^{2} }}{{\omega ( {\omega + i{\Gamma }} )}}$), with $\omega_{p} = 1.37 \times 10^{16}\,\text{Hz}$) and $\Gamma = 1.0027 \times 10^{14}\, \text{Hz}$, while the encasing unit cell is in glass (n = 1.458; in our own simulations we will consider this to be fused quartz, as it fits the definition of glass and its refractive index matches that indicated by Chan et al. at near-infrared frequency^28^).

**Figure 2:**
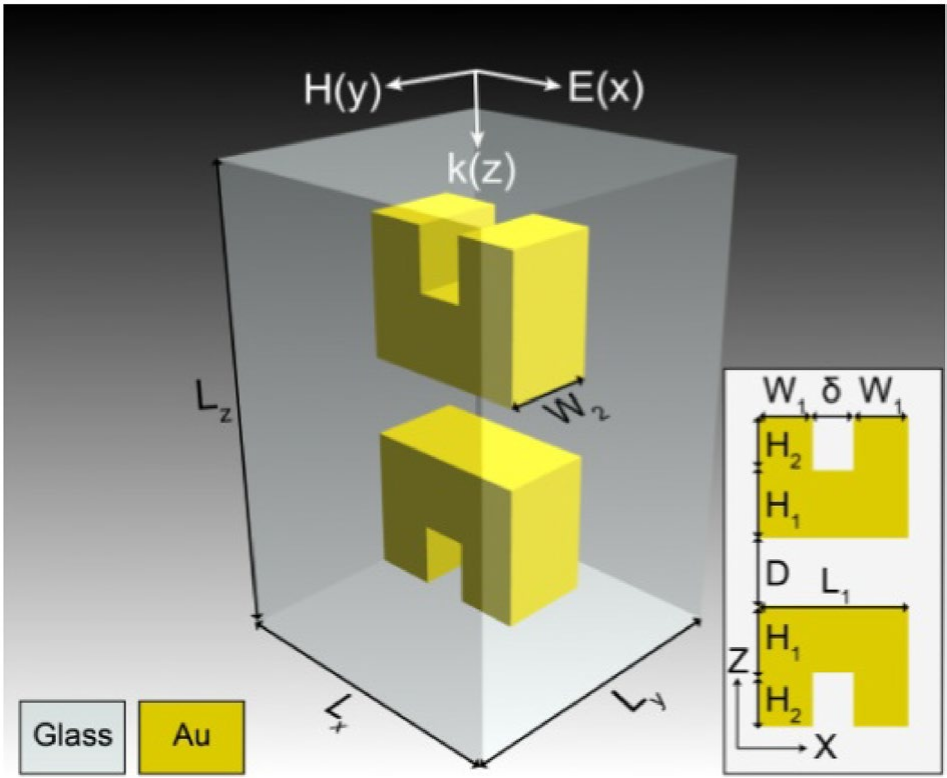
Unit cell of Chan et al.'s upright split-ring pair metamaterial. Dimensions are as follows: *L*_*x*_ = *L*_*y*_ = 200 nm, *L*_*z*_ = 300 nm, *W*_*1*_ = 40 nm, *W*_*2*_ = 60 nm, *H*_*1*_ = 50 nm, *H*_*2*_ = 40 nm, *D* = 50 nm, and δ = 30 nm [Reprinted/Adapted] with permission from^17^ © IOP Publishing.

**Figure 3:**
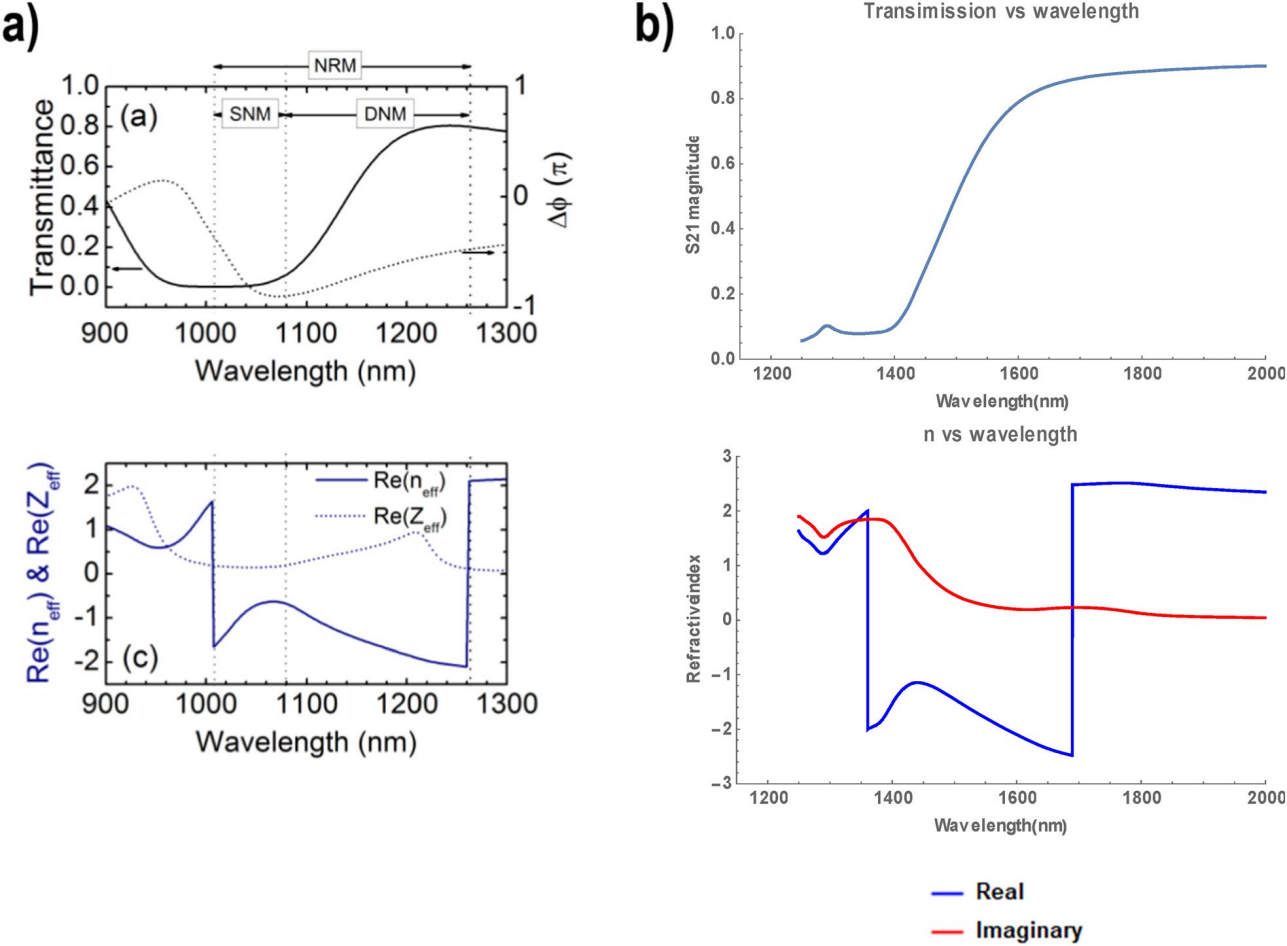
Comparison between Chan et al.'s simulation results^17^ in (**a**) ([Reprinted/Adapted] with permission from^17^ © IOP Publishing) of the upright split-ring pair metamaterial and our own results in (**b**).

A unit cell is represented in Fig. 2. Dimensions are as follows: *L*_*x*_ = *L*_*y*_ = 200 nm, *L*_*z*_ = 300 nm, *W*_*1*_ = 40 nm, *W*_*2*_ = 60 nm, *H*_*1*_ = 50 nm, *H*_*2*_ = 40 nm, *D* = 50 nm, and δ = 30 nm. Chan et al. report a negative refractive index achieved between wavelengths of 1.008 μm and 1.260 μm (238–297 THz). Our simulation, based on Chan et al.'s parameters, conforms in Chan et al.'s response spectral profile, albeit differing by the span of frequency over which negative refraction is observed: we observe negative refraction from 177 to 220 THz. The result can be observed in Fig. 3b. This resonance shift might be a result of the assumption of fused quartz as glass material presented in the previous paragraph, or other parametric differences between our simulation and that of Chan et al. Chan et al. demonstrated the negative refraction of their material by both analysis of the S-parameters using the effective parameter retrieval method, geometrical analysis of the incident rays and by analysis of the dispersion relation of their material. Nowhere in their report do they, however, mention the refractive index jump that occurs at 238 THz (from positive to negative value) and 297 THz (back from negative to positive value) (Fig. 3a).

**Figure 4:**
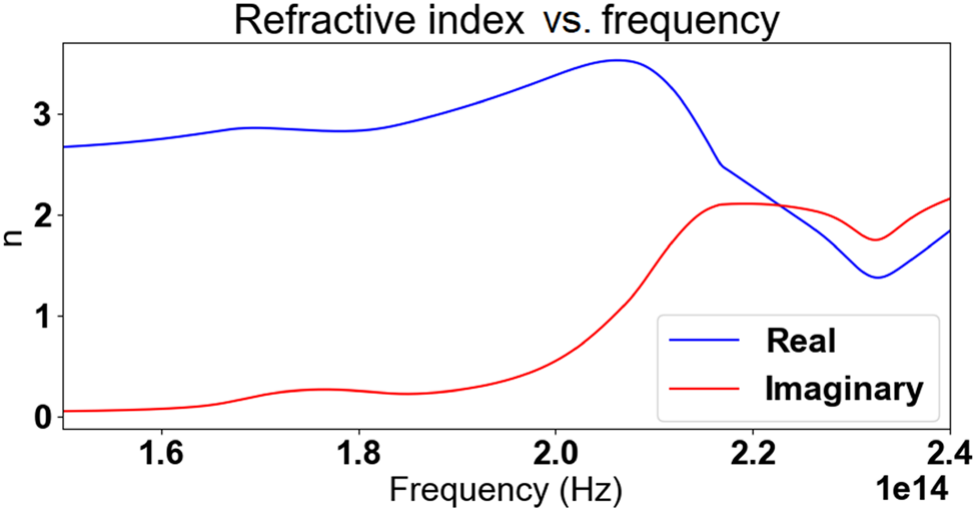
Numerically calculated refractive index of Chan et al.'s metamaterial after correction of the discontinuity.

It is also noteworthy that Chan et al.'s results are the only one among the three we will discuss here, where continuity is enforced—a change in the m-branch index of Eq. (2) at frequencies where the discontinuity can be observed need not take place and the retrieved refractive index can be made continuous across the spectrum of frequencies we're considering here. We chose to disregard this change in m-branch index in Fig. 3 to make the similarity of our numerical simulation to that of Chan et al.'s apparent. When the NRW algorithm is thoroughly applied and continuity of the refractive index is enforced, the refractive index becomes as represented in Fig. 4, and is positive for all frequency values of the considered spectrum.

### Du et al.'s Fishnet three-dimensional metamaterial

**Figure 5:**
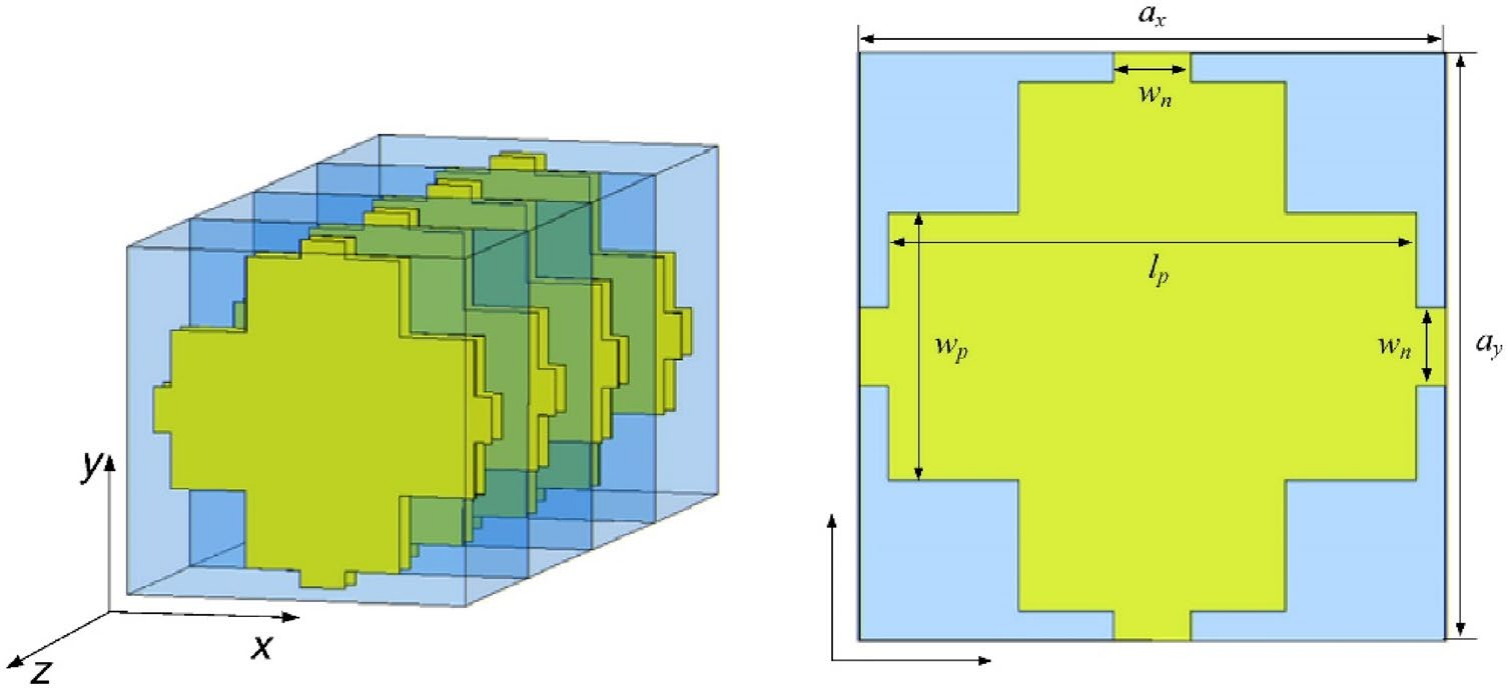
Du et al.'s three-dimensional fishnet metamaterial. Dimensions of the unit cell are *a*_*x*_ = *a*_*y*_ = 90 μm, *a*_*z*_ = 62 μm*, l*_*p*_ = 81 μm, *w*_*p*_ = 41 μm and *w*_*n*_ = 12 μm [Reprinted/Adapted] with permission from^16^ © Elsevier.

Du et al.^16^ proposed a metamaterial operating at low terahertz frequency. Their design consists in a refinement of the isotropic fishnet design^14^ where the central slab is reshaped into a cross (see Fig. 5). As for the original fishnet, the structure is made of layers of gold (Drude model with plasma frequency ω_*p*_ = 1.367 × 10^16^ Hz and collision frequency γ = 4.084 × 10^13^ Hz) in a cross design. Gallium arsenide (GaAs) is sandwiched between the metallic layers of the unit cell. Dimensions of the unit cell are *a*_*x*_ = *a*_*y*_ = 90 μm, *a*_*z*_ = 62 μm*, l*_*p*_ = 81 μm, *w*_*p*_ = 41 μm and *w*_*n*_ = 12 μm.

**Figure 6:**
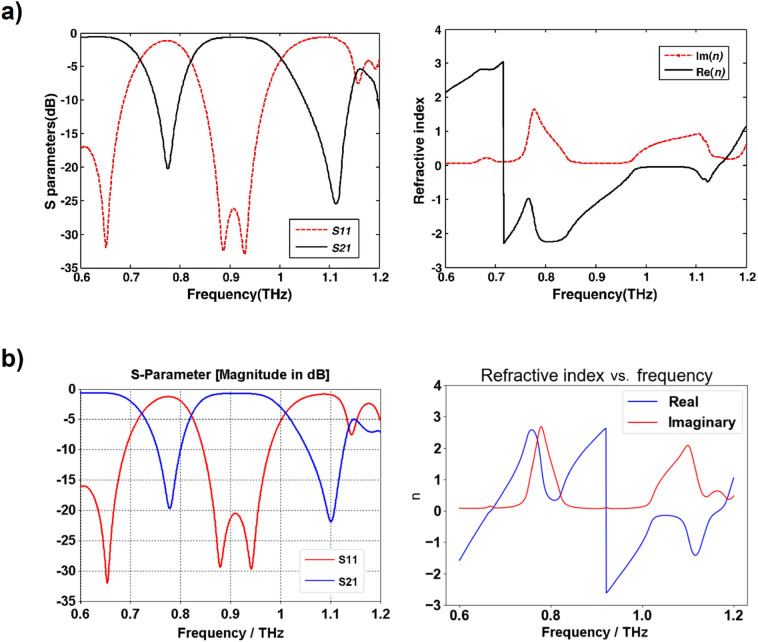
Comparison between Du et al.'s three-dimensional fishnet metamaterial simulation results^16^ in (**a**) ([Reprinted/Adapted] with permission from^16^ © Elsevier) and our own results in (**b**).

Du et al. reported negative refraction in the range 0.72–0.97 THz (Fig. 6a). While the results of our simulation are in good agreement with Du et al.'s when comparing the S-parameters in Fig. 6, the application of the effective parameter retrieval procedure holds seemingly very different results: our application of the procedure leads to a negative refraction appearing only starting at 0.92 THz. A closer inspection reveals however that this is only due to the refractive index jump from positive to negative values, which occurs at an earlier frequency in Du et al.'s analysis. This discrepancy notwithstanding, the profile of the refractive index is very similar if one ignores the discontinuity and only considers the rate of change of the refractive index with respect to the frequency. This rate of change is positive from 0.6 to 0.76 THz, reaches a zero (refractive index local maximum) at 0.76 THz, then turns negative until reaching another zero (refractive index local minimum) at 0.81 THz, and then being positive again. This discrepancy in results highlights the importance of the phenomenon of refractive index discontinuity: Du et al. is able to report a frequency window of negative refraction about five times greater than that one would conclude from our own results. However, no acknowledgment was made of the refractive index discontinuity.

### Paul et al.'s bulk metamaterial

**Figure 7:**
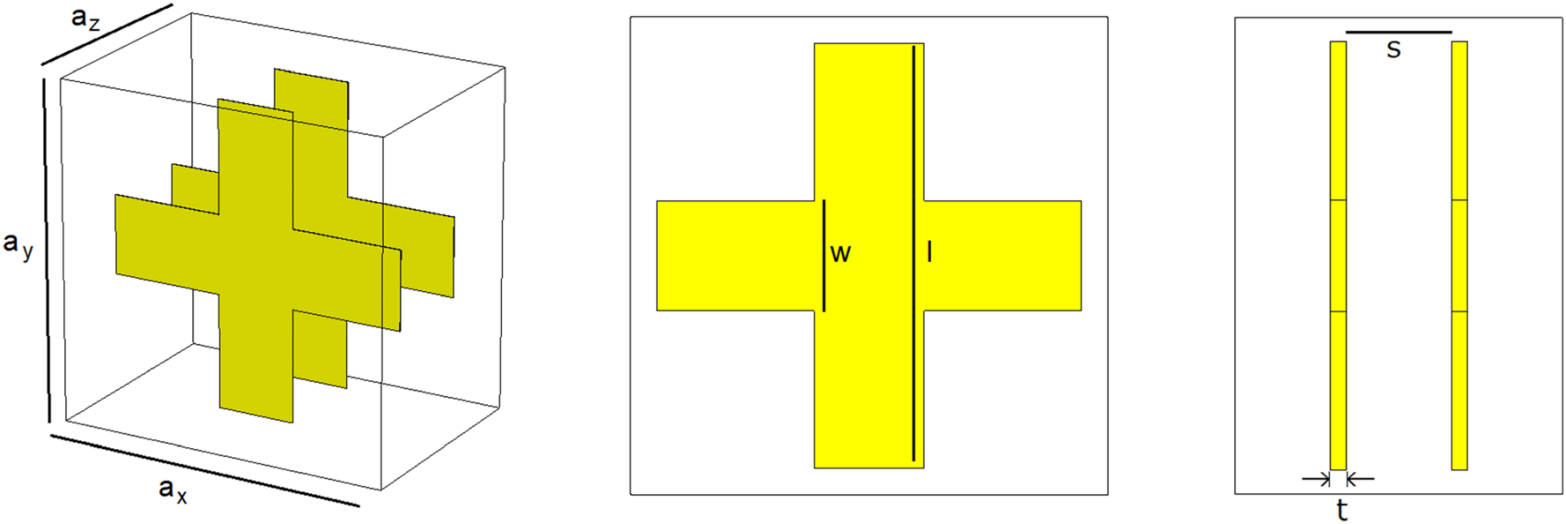
Paul et al.’s bulk metamaterial. Dimensions are as follows: *a*_*x*_ = *a*_*y*_ = 90 μm, *a*_*z*_ = 62 μm*, l*_*p*_ = 81 μm, *w* = 21 μm, s = 9.5 μm and t = 0.2 μm [Reprinted/Adapted] with permission from^27^ © The Optical Society.

**Figure 8:**
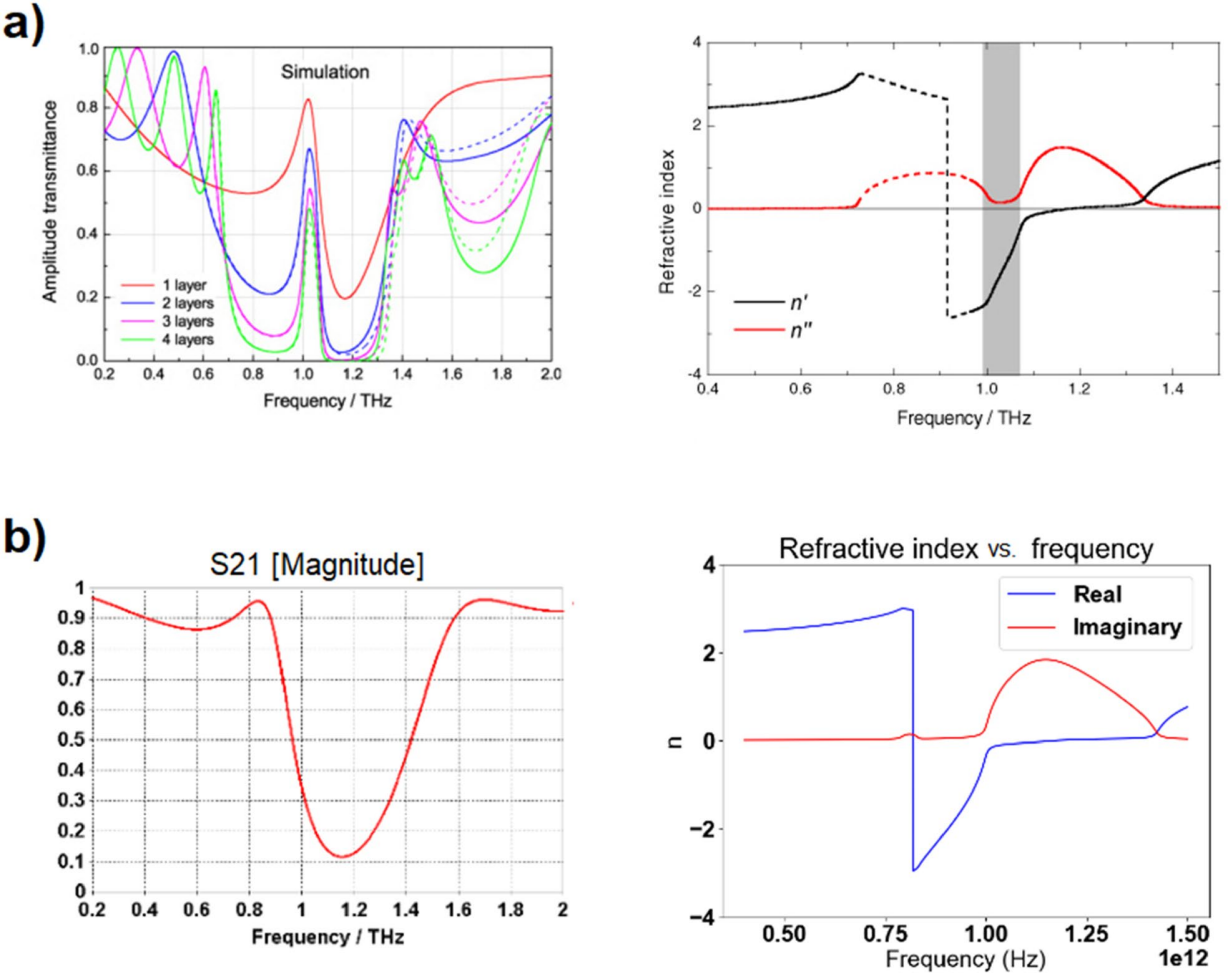
Comparison between Paul et al.'s bulk metamaterial simulation results^27^ in (**a**) ([Reprinted/Adapted] with permission from^27^ © The Optical Society) and our own results in (**b**).

Paul et al.^27^ present a fishnet-like, cross-shaped metamaterial operating at low terahertz frequency. This design is similar to Du et al.'s excepted the presence of arms, which interestingly makes it the only “disconnected" fishnet considered in this article (see Fig. 7). The metallic layers are set to be copper, with Drude permittivity (the Drude characteristic of copper at terahertz frequencies are not specified in Paul et al.'s report; for our own simulation, we set those characteristics to ω_*p*_ = 1.914 × 10^15^ Hz for the plasma frequency and γ = 8.34 × 10^12^ Hz for the collision frequency^29^). Same as with Du et al.'s fishnet, the whole unit cell is permeated with the dielectric, chosen by Paul et al. to be BCB (permittivity of ε = 2.67 and loss parameter tanδ = 0.012 at frequency of interest).Paul et al. reports negative refraction in the frequency window from 0.96 to 1.17 THz. Our own simulation qualitatively agrees with those results, as can be seen in Fig. 8, albeit with a negative refraction starting at the refractive index discontinuity located at 0.82 THz. Paul et al. is the only one to actually acknowledge the discontinuity in their results, stating: “The dashed parts of the curves (Fig. 8a) mark the regions where the half-wavelength inside the medium is smaller than the lattice constant *a*_z_ and the calculated effective parameters are not expected to be reliable"^27^.

## Geometrical study of simulated electromagnetic dynamics as a test for negative refraction

A benchmark was used to investigate the negative refraction (or lack thereof) in metamaterials. This benchmark relies on the geometrical ray formalism of electromagnetic waves, using observations of the behavior of those waves as they are transmitted through objects of determined shapes (mainly, a prism), and comparing those observations to what would be expected should the material be homogeneous and have the claimed refractive index.

The numerical tools at our disposal for the simulation of plane waves as a way to observe the reaction of transmitted waves through the media. This brings a challenge to the analysis of the refractive index using this benchmark: diffraction patterns occur, that, while still allowing an interpretation of the sign of the refractive index, limit the precision of an exact computation of that index. Fortunately, in this analysis we are less concerned by the exact value of the index, than we are about its sign, and the frequency at which the jump from positive to negative refractive index occurs.

**Figure 9:**
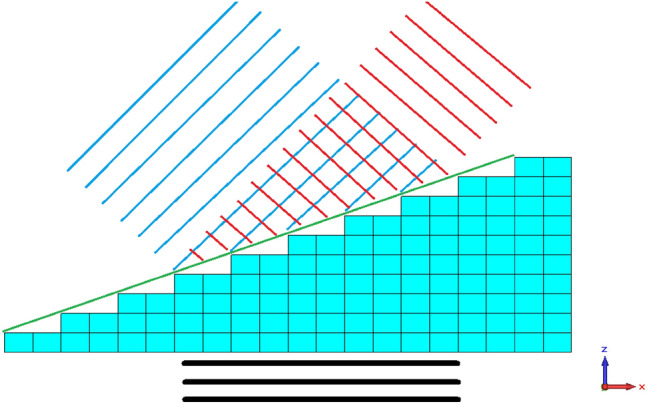
Typical simulation set-up for the prism configuration. Incoming plane wave is symbolized by the black lines. Positively refracted outgoing wave is symbolized by the red lines. The negatively refracted plane wave is symbolized by the blue lines. The green line delineates the idealized upper interface.

The prism configuration is a benchmark that allows to geometrically determining the refractive index of the metamaterial (Fig. 9). A 2D prism is numerically designed using unit cells of the metamaterial one wishes to study, and a plane wave is sent onto a surface of the prism. The direction of the transmitted wave allows the determination of the sign of the refractive index of the metamaterial at that wavelength. Typically, the prism is designed so that the interface of incidence of the plane wave is parallel to the wave front, and the prism is elongated enough to contain the principal front of the wave.

A standard prism is depicted in Fig. 9. The unit cells (cyan) are stacked in a half-pyramid, creating a prism in the XZ plane. A plane wave (wave fronts depicted in black) polarized in the Y direction and propagating towards the Z direction is incident upon the lower interface of the prism, and emerges refracted from the upper interface of the prism. Direction of refraction indicates the sign of the prism's material refractive index: refraction to the left (blue wave fronts) signifies a negative refractive index, while refraction to the right (red wave fronts) signifies a positive refractive index. The ideal upper interfaces pictured in green.

**Figure 10:**
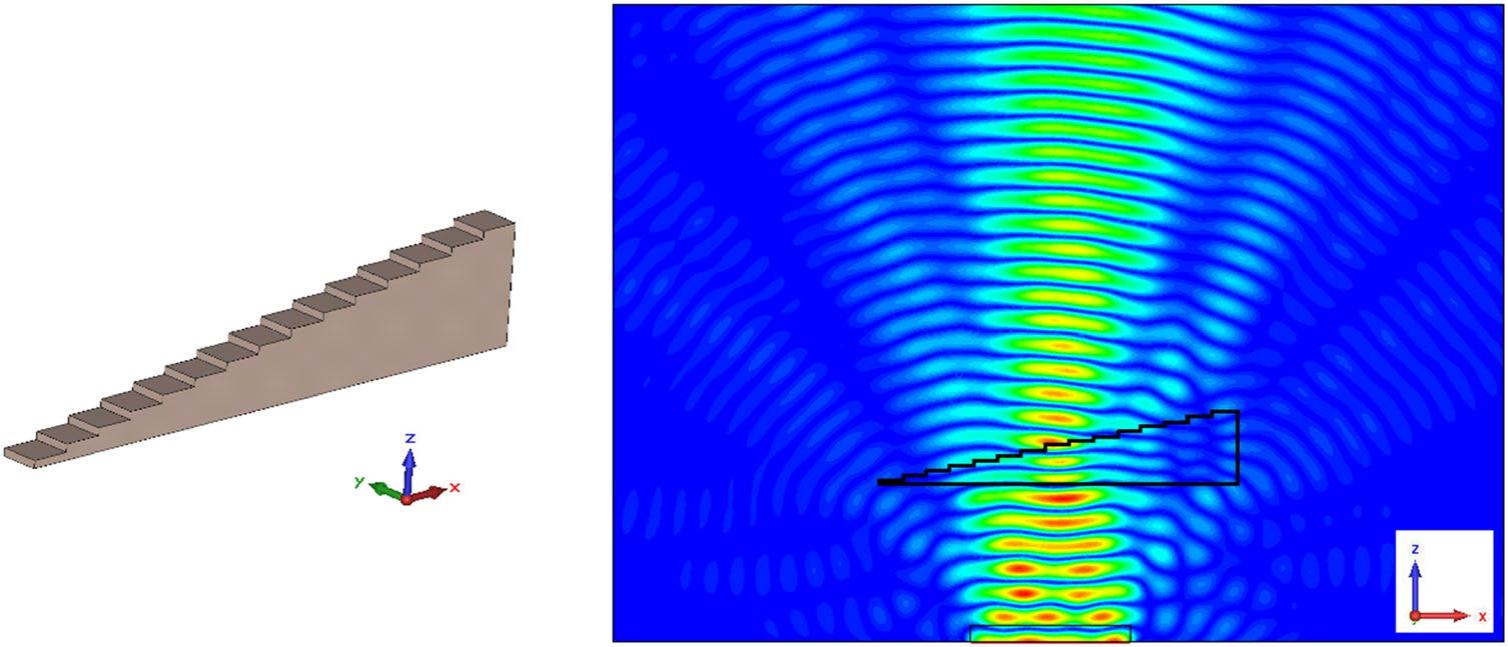
Simulation of the refraction of a plane wave by a Teflon prism. On the left: representation of the simulated Teflon prism, made of 15(15 + 1)/2 blocks of Teflon with dimensions 150 × 150 × 30 μm. On the right: XZ plane section of the absolute value of the electric field generated by a plane wave incident upon the lower interface of the prism. Generated using CST Microwave Studio^30^.

One readily notes a first challenge with this configuration: virtually all metamaterials are constrained by the dimensions of their unit cell, and the development of complex geometrical objects using those metamaterials must take these dimensions into account. For the examples considered in this paper, it is impossible to design a prism with an upper surface that can be considered nanoscopically or even microscopically planar, and that can therefore match the ideal upper interface; the best one can do is to design staircase-shaped prisms (grism) in dimensions such that a negligible amount of scattering occurring due to the local nonnuniformities of the upper surface. However, practically speaking this staircase shape does not lead to significant scattering if we constrain the slope by an increment of one unit cell's depth as we move laterally towards the positive X. An example demonstrating this can be found in Fig. 10, representing a prism with similar geometry as that of Fig. 9, made of $\frac{{15 ( {15 + 2} )}}{2}$ homogeneous blocs of Teflon with dimensions 150 × 150 × 30 μm. An electromagnetic wave of frequency 1 THz, polarized along the Y-axis and propagating towards the positive Z, is sent upon the inferior interface of the prism. One clearly distinguishes the main refracted wave leaving the upper interface of the prism towards the right (as expected from a positive refraction), as well as secondary waves of lower intensities refracted right and left of the main wave, due to diffraction effects of the input port.

### Analysis of Chan et al.'s metamaterial

**Figure 11:**
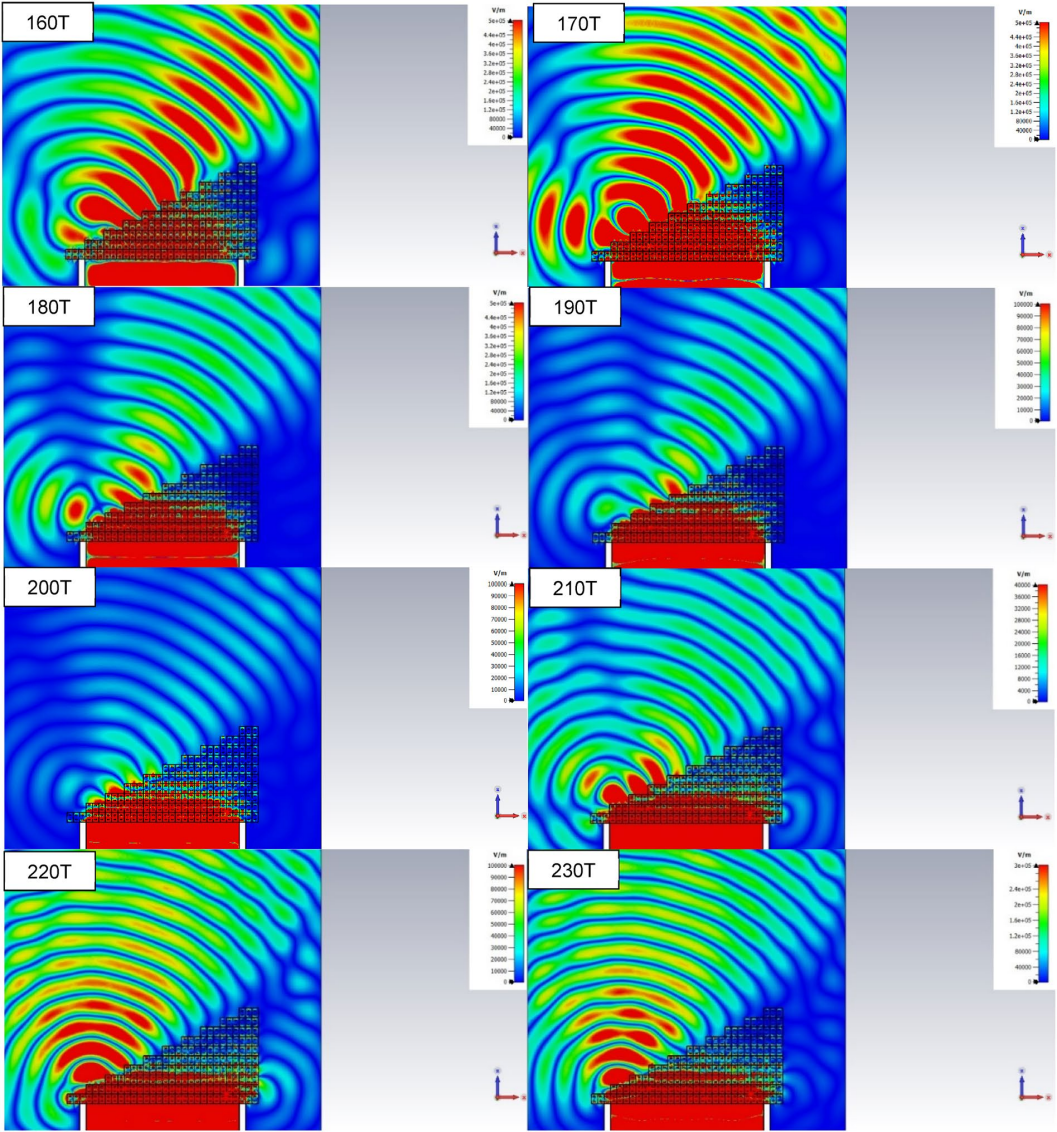
Absolute value of the electric field within and around a prism made of Chan et al.'s metamaterial for various frequencies when a plane wave is incident upon it, based on a numerical simulation. The shape of the prism is delineated for clarity for the first frequency. Generated using CST Microwave Studio^30^.

Figure 11 shows a simulation of a plane wave incident upon a prism made of unit cells of Chan et al.'s metamaterial. The absolute value of the electric field is represented within and around the prism (the shape of which is explicit in black for the first frequency).

Comparing those results to those obtained by (mis-)use of the NRW algorithm, no negative refraction is observed at frequencies where expected from Fig. 3b. Instead, positive refraction is observed at frequencies 160–180 THz. At those frequencies, transmission drops to levels that prevent any analysis of the refracted plane wave. Between frequencies of 210–230 THz, strong reaction, confirmed to happen at those frequencies by numerical simulation of a single unit cell, causes the incident plane wave to “escape" laterally from the lower interface of the prism. These results confirm the necessity of enforcing continuity of the refractive index whenever possible. In the last two cases however, discontinuity has to occur, as we will show.

### Analysis of Du et al.'s fishnet three-dimensional metamaterial

**Figure 12:**
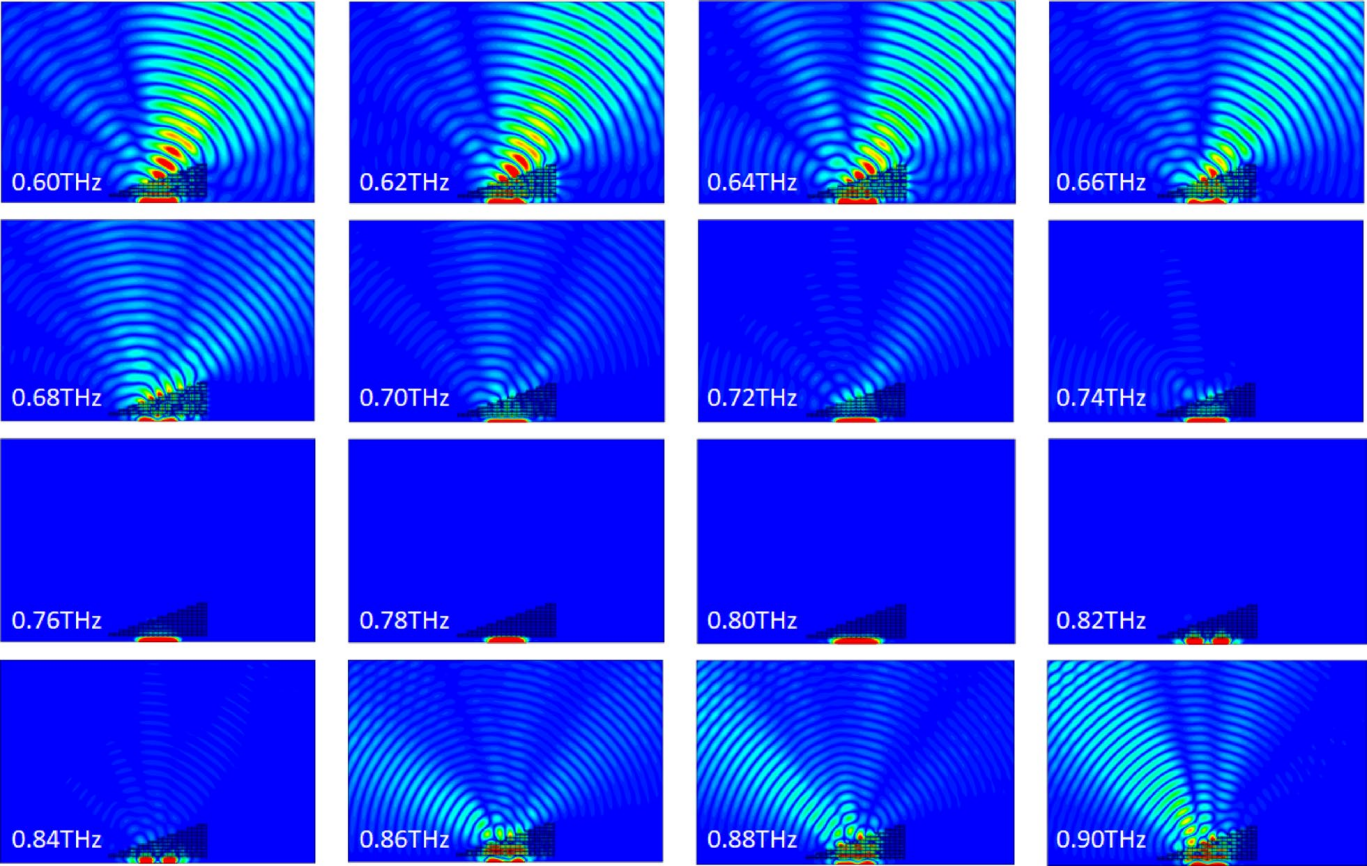
Absolute value of the electric field within and around a prism made of Du et al.'s metamaterial for various frequencies when a plane wave is incident upon it, based on a numerical simulation. Generated using CST Microwave Studio^30^.

Figure 12 shows a simulation of a plane wave incident upon a prism made of unit cells of Du et al.'s metamaterial. The absolute value of the electric field is represented within and around the prism.

**Figure 13:**
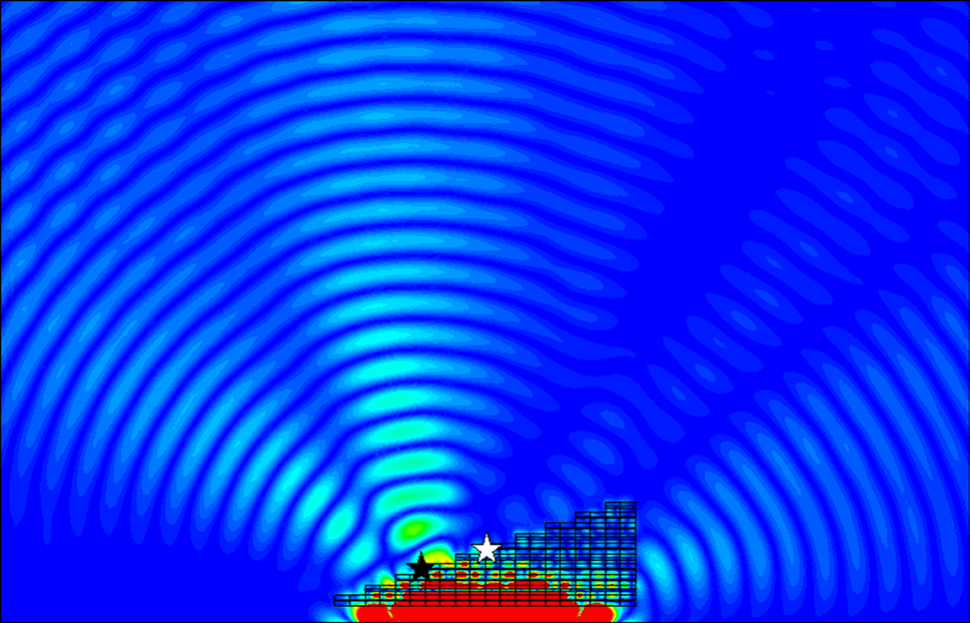
Absolute value of the electric field within and around a prism made of Du et al.'s metamaterial when a plane wave of frequency 0.8 THz is incident upon it, based on a numerical simulation. The white star indicates the center of the expected emerging plane wave, and the black star the position of the actual center of the emerging plane wave. Generated using CST Microwave Studio^30^.

Positive refraction is observed from 0.6 to 0.72 THz, after which a drop in transmission is observed (and confirmed in the numerical results presented in Fig. 6, with a transmission's amplitude under 0.60 in the span 0.73–0.82 THz and a minimum of 0.10 reached at frequency 0.78 THz), rendering any observation impossible. Negative refraction is observed at 0.86 THz transmission rebounds. Enhancement of the electric field is done by rescaling of the color gradient. However, because of the high loss of the material only the part of the plane wave that crosses a small thickness of prism emerges from the upper interface, skewing the results (see Fig. 13 for an illustration of the effect; the white star is the desired center of the wave emerging from the upper interface, and the black star the observed center). Thus, this analysis does not allow for an exact determination of the frequency at which the refractive index passes from positive to negative, but allows to determine a region of uncertainty. In particular, it points out at an error in our application of the NRW algorithm, as our computation of the refractive index predicted a negative refraction only starting frequency 0.92 THz. Details about the origin of this error will be covered later in this article.

### Analysis of Paul et al.'s fishnet three-dimensional metamaterial

**Figure 14:**
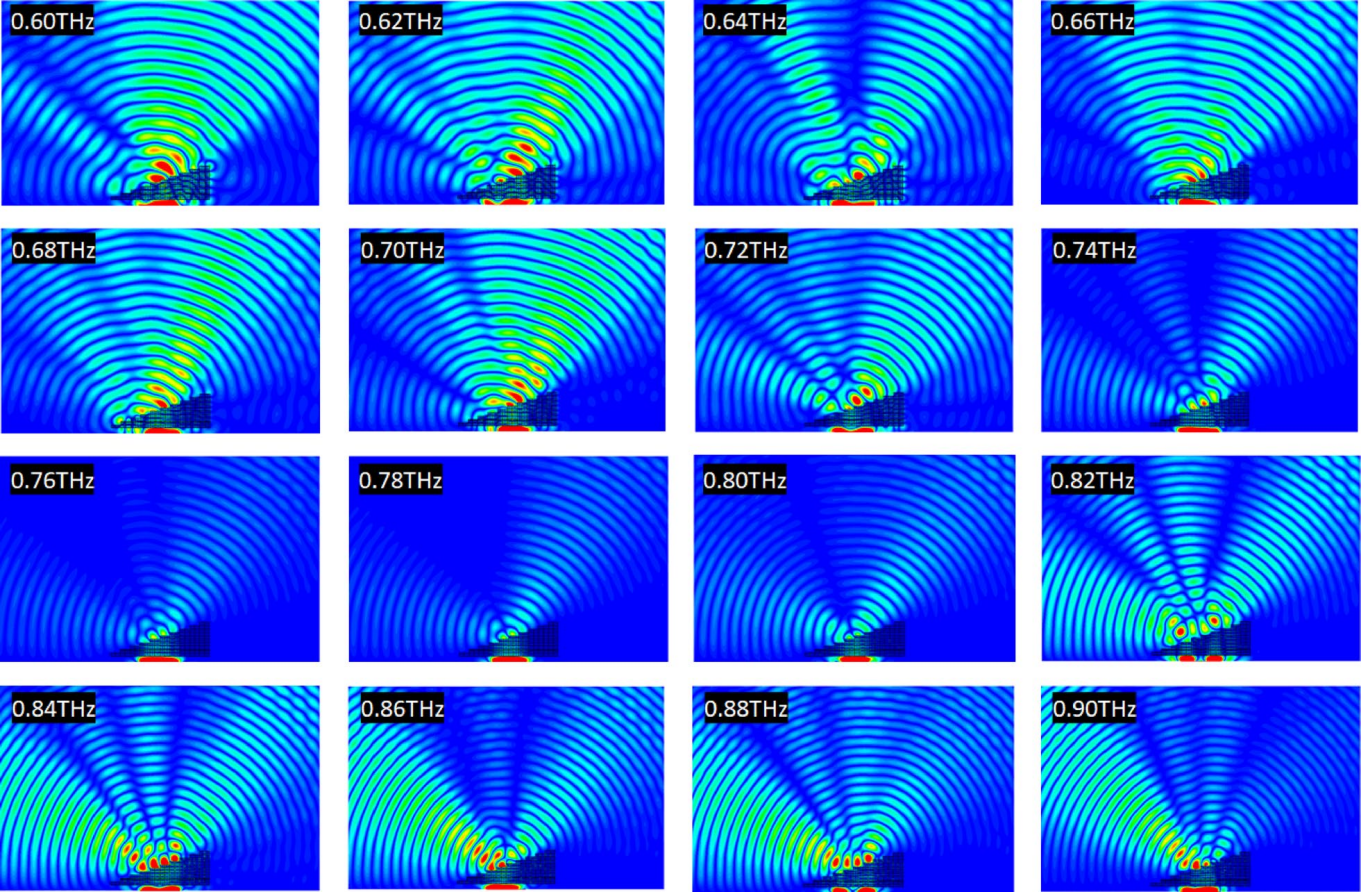
Absolute value of the electric field within and around a prism made of Paul et al.'s metamaterial for various frequencies when a plane wave is incident upon it, based on a numerical simulation. Generated using CST Microwave Studio^30^.

Figure 14 shows a simulation of a plane wave incident upon a prism made of unit cells of Paul et al.'s metamaterial. The absolute value of the electric field is represented within and around the prism.

Positive refraction is observed from 0.6 to 0.74 THz. Starting 0.86 THz, a clear negative refraction is observed. Between 0.74 and 0.86 THz, an interesting phenomenon occurs: the emerging positively refracted wave front gradually diminishes in intensity while a negatively refracted wave front gradually increases in intensity, with a seemingly equal wave intensity observed at frequencies 0.80–0.82 THz, where the plane wave seems to split at the upper interface.

Regardless of the ambiguity observed between frequencies 0.74–0.86 THz, our observation allows us to extend the reported frequency spectrum over which negative refraction occurs (the grey zone in Fig. 8a) from starting at 0.96 THz to starting at 0.86 THz.

## Numerical and theoretical investigation of the refractive index discontinuity

### Modulo-2π transmission phase jump as a cause for refractive index discontinuity

So far, we have observed two cases of discontinuity: one that turned out to be a misapplication of the NRW algorithm (i.e. Chan et al.'s metamaterial), and one that turned out to be a shift in refractive index verified by numerical simulation (i.e. Paul et al.'s and Du et al.'s metamaterials).

**Figure 15:**
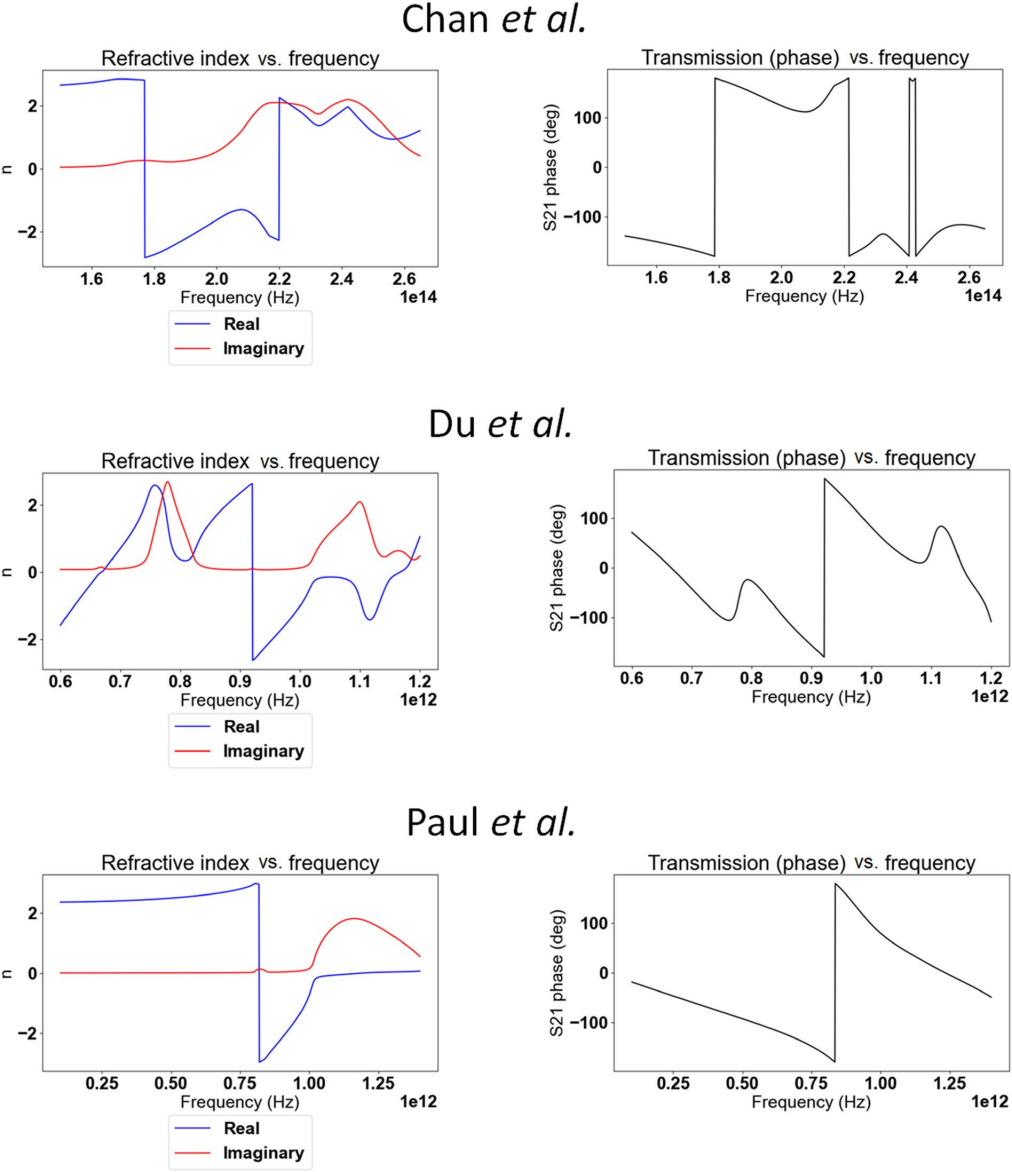
Comparison of numerical results of, from top to bottom, Chan et al.'s, Du et al.'s and Paul et al.'s metamaterials refractive index (left) and transmission phase (right). To note, Chan et al.'s refractive index is represented as obtained by misuse of the NRW algorithm.

Let us first investigate the case of Chan et al.'s metamaterial. Figure 15 shows, side by side, the numerically retrieved transmission phase of the metamaterials, as well as the numerically retrieved refractive index with, in the case of Chan et al., misuse of the NRW algorithm (i.e. non enforcement of the continuity requirement). One readily notices that refractive index discontinuities occur at the exact same frequencies as a “modulo jump" of the transmission phases: the latter discontinuities are expected from our numerical simulation software, which constrains the value of phase to the range [− 180°; + 180°].

This phase discontinuity directly affects the NRW algorithm. Indeed, if one follows the dependence of n on S21 (see Eq. 2), an expression of the following form can be established for the real part of n, with the arc cos rewritten as a logarithm equivalent:

3 $$ Re ( n ) = \frac{1}{{k_{o} d}}\left[ {{\text{Im}}\left( {{\text{ln }}\Delta} \right) + 2{\text{m }}\pi} \right] $$

with the full expression of D as:

4 $$\begin{aligned} {\Delta } &= \frac{{1 - S_{11}^{2} + S_{21}^{2} }}{{2S_{21} }} \pm i\sqrt {1 - \left( {\frac{{1 - S_{11}^{2} + S_{21}^{2} }}{{2S_{21} }}} \right)^{2}  }\\ &= \frac{1}{{2S_{21} }}\left\{ {1 - S_{11}^{2} + S_{21}^{2} }  \pm { \sqrt {\left[ {\left( {S_{11}^{2} - S_{21}^{2} } \right)^{2} - 1} \right]\left[ {\left( {S_{11}^{2} + S_{21}^{2} } \right)^{2} - 1} \right]} } \right\} = \frac{{\Lambda }}{{2S_{21} }} \end{aligned} $$

Replacing this expression in Eq. (3), we obtain:

5 $$ Re\left( n \right) = \frac{1}{{k_{o} d}}\left[ {{\text{Im}}\left( {{\text{ln}}\frac{{\Lambda }}{{2{\text{S}}_{21} }}} \right) + 2m\pi } \right] = \frac{1}{{k_{o} d}}\left[ {{\text{Im}}\left( {{\text{ln}}\frac{{\Lambda }}{{2{\text{S}}_{21} }}} \right) - {\text{arg}}\left[ {S_{21} } \right] + 2m\pi } \right] $$

In other words, the real part of the refractive index possesses a term that is directly proportional to the transmission phase. One notices a correspondence between this result and Fig. 15: the jumps in transmission phase and refractive index are opposite in direction, one going up when the other goes down, justified by the negative sign of the arg[S21] term in Eq. (5). Furthermore, these phase jumps correspond exactly to a m-branch jump, since incrementing the m-branch index by one is equivalent to dropping the transmission phase by 360 degrees (2π). This is therefore proof that close monitoring of the m-branch index is able to solve mistaken discontinuities in the application of the NRW algorithm, such as with Chan et al.'s.

One would be tempted to formulate a rule to always accompany a transmission phase jump by a corresponding change in m-branch index, thereby always enforcing continuity. This, however, works only in the case where enforced continuity is validated by numerical simulations such as tests carried by prism refraction. In the cases of the metamaterials presented by Du et al. or Paul et al., we have observed that a refractive index jump is necessary to justify the observed transition from positive to negative refraction.

### Impact of refractive index discontinuity on the propagating wave

As seen in Eq. (5), a m-branch index jump leads to an increment/decrement of $2\pi /k_{o} d$ of the real part of the refractive index. This affects several variables related to the propagation of waves within the medium.

Assuming the metamaterial has interfaces with vacuum, where a plane wave propagates with wavelength λ_o_, the wavelength within the metamaterial becomes λ_meta_ = λ_o_/Re(n). The subtraction of a $2\pi /k_{o} d$ term to the real part of the refractive index leads to a new wavelength within the metamaterial:

6 $$ \lambda_{meta}^{^{\prime}} = \frac{{\lambda_{o} }}{{Re\left( n \right) - \frac{2\pi }{{k_{o} d}}}} = \frac{1}{{\frac{1}{{\lambda_{meta} }} - \frac{1}{d}}} $$

This affects the propagating wave phase that becomes (assuming propagation in the z direction):

7 $$ \begin{aligned} kz - \omega t &= \frac{{2\pi \left( {z - \frac{ct}{n}} \right)}}{{\lambda_{meta} }} \\ &\quad \to 2\pi \left( {z - \frac{ct}{{n - \frac{{\lambda_{o} }}{d}}}} \right)\left( {\frac{1}{{\lambda_{meta} }} - \frac{1}{d}} \right) = z\left( {k - \frac{2\pi }{d}} \right) - \omega t \end{aligned} $$

One notices the term associated with spatial propagation has become negative (bearing in mind that d ≪ λ_meta_), as expected from the ip in wave vector k direction that occurs when passing from positive refraction to negative refraction (i.e. the wave vector k goes in the same direction as the Poynting vector in regular materials, but in the opposite direction in metamaterials).

**Figure 16:**
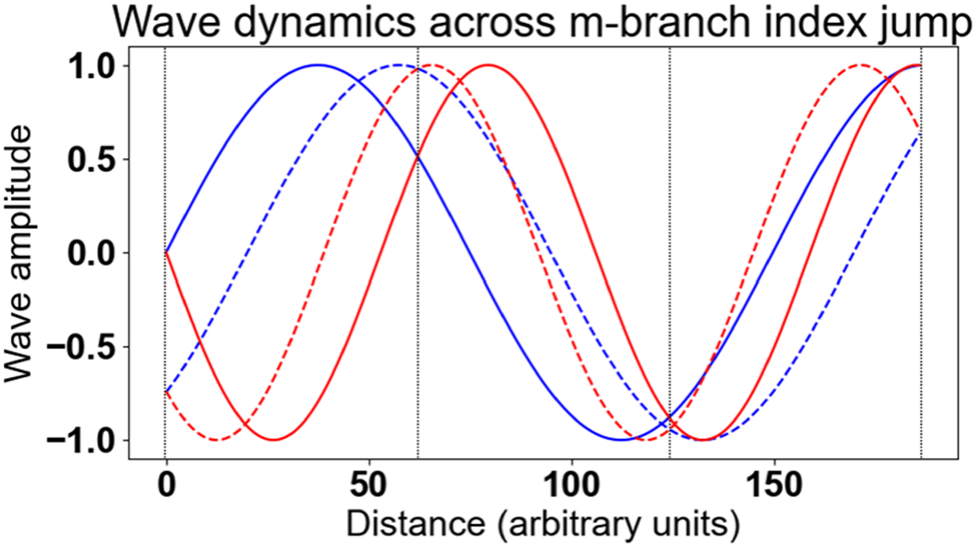
Example of the dynamics of waves across a m-branch index jump. Wave propagation is represented as, for a given color, solid line for the wave position at an instant t and dashed line for the wave position at an instant t + ε. Blue wave represents propagation in a medium with positive refractive index (i.e. wave fronts moving towards the positive z), Red wave represents propagation in a medium with negative refractive index after m-branch index jump (i.e. wave fronts moving towards the negative z). Vertical dashed black lines indicate the boundaries of each unit cell, where the phase of blue and red waves match at any time.

Equation (7) reveals that the phases at any boundary of a stack of metamaterial unit cells remain unchanged after a refractive index jump, specifically being incremented by a multiple of 2π: considering a stack of thickness md with *m* a signed integer (including 0) and d the thickness of a single unit cell, the phase at *z* = *md* passes, as the refractive index jumps to a lower m-branch index, from *mdk − *ω*t* to *mdk − 2 m*π* − *ω*t*. However, the sine wave within the metamaterial has been flipped," as shown in Fig. 16. The angular frequency ω changes neither in value nor in sign, so that the wave internal to the metamaterial has now a negative phase velocity.

These considerations show that, while the topology of the effective wave has changed within the metamaterial, the dynamics of the phases remain unchanged at any time at the metamaterial's boundaries. Bearing in mind that the effective wave described by the effective parameters resolved by application of the NRW algorithm is just a convenient model of the metamaterial's properties, and not and actual description of the electromagnetic dynamic within, so that no actual “wave flip" occurs, the refractive index discontinuity is therefore entirely compatible with upon which a plane wave is incident.

## Conclusion

In this article, we have analyzed three instances of metamaterials reported in the literature who display a discontinuity on the frequency spectrum. We performed numerical simulations of metamaterial unit cells with similar parameters as those described in those reports to ascertain a faithful reproduction of the report's research, and match the results of our numerical simulations with those of those reports. We then performed a numerical simulation of a prism constituted of the aforementioned metamaterials, upon which was incident a plane wave of frequency of resonance of the metamaterial. We were thus able to identify one metamaterial that did not display negative refraction, and two were. In particular, we were able to identify that Paul et al.’s metamaterial displays a beam-splitting phenomenon that warrants further research.

We identified further the cause leading to the emergence of the refractive index discontinuity, and developed a mathematical formalism to justify the connection between this discontinuity and the 2π modulo correction of the transmission phase. We finally demonstrated that while this discontinuity leads to a change of the behavior of the effective electromagnetic wave within the metamaterial, the behavior of the electromagnetic wave external to the metamaterial remains unchanged; as this discontinuity does not affect the phase of the wave at the metamaterial's boundaries.

## Data Availability

The datasets generated during and/or analyzed during the current study are available from the corresponding author on reasonable request.
